# Retinal Pigmented Epithelial Cells Cytotoxicity and Apoptosis through Activation of the Mitochondrial Intrinsic Pathway: Role Of Indocyanine Green, Brilliant Blue and Implications for Chromovitrectomy

**DOI:** 10.1371/journal.pone.0064094

**Published:** 2013-05-10

**Authors:** Fernando M. Penha, Marianne Pons, Elaine Fiod Costa, Nilana Meza Tenório Barros, Eduardo B. Rodrigues, Emmerson Badaró Cardoso, Eduardo Dib, Mauricio Maia, Maria E. Marin-Castaño, Michel Eid Farah

**Affiliations:** 1 Departamento de Oftalmologia, Instituto da Visão (IPEPO), Universidade Federal de São Paulo, São Paulo, SP, Brazil; 2 Bascom Palmer Eye Institute, University of Miami, Miami, Florida, United States of America; University of Tennessee, United States of America

## Abstract

**Purpose:**

To investigate the *in vitro* effect of four vital dyes on toxicity and apoptosis in a human retinal pigment epithelial (RPE) cell line.

**Methods:**

ARPE-19 cells were exposed to brilliant blue (BriB), methyl blue (MetB), acid violet (AcV) and indocyanine green (ICG). Balanced salt solution was used as control. Five different concentrations of each dye (1, 0.5, 0.25, 0.05 and 0.005 mg/mL) and two exposure times (3 and 30 min) were tested. Cell viability was determined by cell count and MTS assay and cell toxicity by LDH assay. Real-time PCR and Western blotting were used to access the apoptosis process.

**Results:**

ICG significantly reduced cell viability after 3 minutes of exposure at all concentrations (p<0.01). BriB was safe at concentrations up to 0.25 mg/mL and MetB at concentrations up to 0.5 mg/mL, while AcV was safe up to 0.05 mg/ml, after 3 minutes of exposure. Toxicity was higher, when the cells were treated for 30 minutes. Expression of Bax, cytochrome c and caspase-9 was upregulated at the mRNA and protein level after ICG exposure, while Bcl-2 was downregulated. AcV and MetB were similar to control. However, BriB resulted in upregulation of Bcl-2, an antiapoptotic protein.

**Conclusions:**

The safest dye used on RPE cells was MetB followed by BriB and AcV. ICG was toxic at all concentrations and exposure times tested. Moreover, ICG was the only dye that induced apoptosis in ARPE-19 cells. BriB significantly increased Bcl-2 protein levels, which might protect against the apoptosis process.

## Introduction

The internal limiting membrane (ILM) is a semi-transparent inner layer of the retina composed of collagen type IV measuring approximately 1–10 microns in thickness [1]. The ILM is directly involved in the pathophysiology of macular hole due to tangential forces applied to the retinal surface. Thus, removal of the ILM is an important step for anatomical and functional success in macular hole and other macular surgeries [1]–[3].

Because of its anatomical characteristics, the identification of the ILM during surgery is a difficult step in the surgical procedure. Therefore, the use of dyes to identify structures during vitreoretinal surgery, “chromovitrectomy,” has become a popular technique in recent years [4]. Initially, Burk and colleagues (2000) described the use of indocyanine green (ICG) as a dye for ILM [5]. Currently, there is a general consensus that the removal of the ILM assisted by ICG is technically easier. However, several studies have demonstrated toxicity to the retinal pigment epithelium (RPE) and neurosensory retina, as well as cases of optic nerve atrophy, after the use of ICG [2], [6]–[9]. Due to the toxic potential of ICG, other alternatives have emerged for staining the ILM.

Trypan blue (TB) and brilliant blue (BriB) have been developed as second generation dyes for chromovitrectomy [10]–[14]. TB demonstrated a lower toxicity profile to RPE cells and retinal tissue when compared to ICG, but it is not a good dye for acellular membranes, such as the ILM [7], [15], [16]. BriB was recently released on the European market in a concentration of 0.25 mg/ml – Brilliant Peel^TM^ (DORC, The Netherlands). This presentation of the dye was shown to provide a good staining capacity to the ILM and was not toxic in experimental studies and case series in humans [17]. However, our group showed a selective toxicity to photoreceptors related to BriB after intravitreal injection in rabbit eyes and RPE changes on fluorescein angiography in accidental subretinal dye injection in humans [18]–[20].

ICG, TB and BriB are currently used in chromovitrectomy on the basis of our familiarity with their applications in ophthalmology. However, a dye with little toxicity, preferably inert, and with a good affinity to the ILM is yet to be found. The aim of this study was to provide a detailed *in vitro* toxicity investigation of two new dyes, namely methyl blue (MetB) and acid violet (AcV), and compare it to BriB and ICG, which are in clinical use. Five dye concentrations (1, 0.5, 0.25, 0.05 and 0.005 mg/ml) and two exposure times (3 and 30 min) were examined.

The evaluation of apoptosis in retinal toxicity studies of dyes has become an important issue, since it was shown that residual ICG can be found months after surgery [21]. Therefore, in the present study we also evaluated the link between cell toxicity and apoptosis in ARPE-19 cells exposed to these vital dyes.

## Materials and Methods

### Compounds

The dyes ICG, BriB, MetB and AcV and cell culture reagents were obtained from Sigma-Aldrich (Munich, Germany). Balanced salt solution – BSS (BSS) was obtained from Alcon Laboratories (Fort Worth, TX, USA). MTS CellTiter 96 Aqueous One Solution Cell Proliferation Assay was purchased from Promega (Madison, WI, USA). LDH-Cytotoxicity assay kit was purchased form Abcam Inc (Cambridge, MA, USA). Primary antibodies Bax, BcL-2, cytochrome c, and caspase-9, were purchased from EMD Millipore Corporation (Billerica, MA, USA). The secondary antibodies were obtained from Santa Cruz Biotechnology (Santa Cruz, CA, USA).

### Dye preparation

Initially, 5 mg of each dye were measured using an analytical balance (Mettler-Toledo Inc., Columbus, OH, USA) and dissolved in sterile BSS to obtain stock solutions. ICG required a different form of preparation; it was first diluted in distilled water as recommended by the manufacturer. Subsequent dilutions were performed with sterile BSS to obtain final concentrations of 1, 0.5, 0.25, 0.05 and 0.005 mg/ml. This serial dilution was made to evaluate a wide range of concentrations that could be present in the surgical field due to variations in dilution technique and dye injection (air or fluid filled vitreous cavity).

Afterwards, the pH and osmolarity of each dye solution were determined using a previously calibrated pH meter and osmometer (Advanced Instruments Inc., Norwood, MA, USA). These measurements were made to minimize deleterious conditions related to unexpected variations in pH and osmolarity, which could have an influence on the toxicity found in the experiments.

### Cell-viability assay

All experiments were performed using the immortalized human retinal pigment cell line ARPE-19 (American Type Culture Collection, Manassas, VA, USA), a well-established model to test the safety of vital dyes in RPE cells. The rationale for using this cell line in dye toxicological studies is that during macular hole surgery the dye could come in direct contact with the RPE through the macular hole. Another reason is that the dye can penetrate trough retinal layers and cause RPE toxicity as well. Therefore, a meticulous study of this cell layer is very important in these pre-clinical toxicity evaluations.

ARPE-19 cells were grown in Dulbecco's Modified Eagles Medium/Ham's F-12 (DMEM/F12; Gibco, Carlsbad, CA, USA) (1∶1 vol/vol) medium supplemented with 10% fetal bovine serum (FBS), 1mM L-glutamine, 100 g/ml penicillin/streptomycin, and 0.348% Na_2_CO_3_ in a 5% CO_2_ humidified air incubator at 37°C. Cells were used between passage 5 and 8. For experiments, cells were seeded at a concentration of 5×10^3^ cells/well in 96-well, flat-bottom tissue culture plates in 200 microliters of culture medium and grown for 24 hours before the experiments in a 5% CO_2_ humidified incubator at 37°C. Subsequently, the cell culture medium was removed and the cells washed three times with BSS. The cells were then incubated with MetB, BriB, AcV or ICG (1, 0.5, 0.25, 0.05 and 0.005 mg/ml) for 3 or 30 minutes. After incubation, cells were washed three times with 200 µl of phosphate buffered saline (PBS). The number of surviving cells was measure by cell count (Coulter ZI cell counter; Beckman Coulter, Hialeah, FL, USA) and by MTS (a tetrazolium salt) assay (Cell Titer 96 AQueous One Solution kit; Promega, Madison, WI, USA). The results for MTS were obtained by measuring absorbance at 490 nm with an ELISA plate reader (Bio-Rad, Hercules, CA, USA). All experiments were performed in quadruplicate and repeated three times.

The rationale of these two time exposures, 3 and 30 minutes, was to simulate an acute exposure to these dyes that occurs during vitreoretinal surgery (3 min) and also a prolonged exposure that could happen if the dye is not entirely washed out from the vitreous cavity (30 min).

### Cytotoxicity assay

Lactate dehydrogenase (LDH) is a stable cytosolic enzyme that is released in the culture medium upon cell lysis and the released LDH is measured colorimetrically with maximum absorbance read at 440 nm [22]. LDH catalyzes the readily reversible reaction involving the oxidation of lactate to pyruvate, forming NAD+ from NADH and the determination of lactate dehydrogenase is based on the detection of NADH in the reaction [22]. The treatment protocol was as mentioned for cell viability assay and the conditioned medium alone was taken for LDH leakage assay using an ELISA kits following the manufacturer's instructions. To 1 ml of buffered substrate, 0.1 ml of conditioned media was added and kept in a water bath at 37°C. Next, 0.2 ml of NAD+ solution was added, mixed gently and incubated at 37°C for 15 min. Next, 1 ml of DNPH reagent was added and the mixture incubated for another 15 min. Finally, 10 ml of sodium hydroxide (0.4 N) were added and after 1–5 min, the absorbance was read at 440 nm. Standards were also run simultaneously and treated as for assays with sodium pyruvate to prepare the standard graph. The amount of color formed is proportional to the number of lysed cells. LDH activity  =  OD of unknown/OD of known × standard concentration  =  μg of Lactate released/ml of conditioned media.

### Apoptosis assay

#### Background

Recent studies have delineated one key mechanism responsible for initiating the executioner phase of apoptosis. Early in the process, mitochondria releases cytochrome c [23], which upon entry into the cytosol forms a complex with another molecule, Apaf-1 [24], [25] and the unprocessed (and inactive) proform of a caspase, caspase 9 [26].

It is widely recognized that apoptosis is mediated by Bax cascade through mitochondrial stress [27]. Bax is a pro-apoptotic Bcl-2-family protein that resides in the cytosol and translocates to mitochondria upon induction of apoptosis [28]. Recently, Bax has been shown to induce cytochrome c release and caspase activation *in vivo* [29] and *in vitro* [30]. In contrast, the antiapoptotic protein Bcl-2 has been shown to be capable of blocking spontaneous cytochrome c release in cell-free extracts and in cells treated with apoptosis-inducing agents [31].

We therefore examined the modulation of apoptotic proteins (Bax, cytochrome c, and caspase-9) and antiapoptotic protein Bcl-2 by MetB, AcV, BriB and ICG in ARPE-19 cells exposed to 0.05 mg/ml dyes for 3 minutes, which is the concentration and time mostly used in vitreoretinal surgery.

#### Real-time PCR

ARPE-19 cells were cultured as described above and exposed to 0.05 mg/ml ICG, BriB, AcV or MetB for 3 minutes. BSS was used as the control. Afterwards, the dye was removed and each well was washed three times with 200 microliters of PBS. RNA was extracted using the TRI reagent (Sigma-Aldrich, St Louis, MO, USA). RNA concentrations were measured spectrophotometrically (Nanodrop 2000, ThermoScientific, Waltham, MA, USA), and the quality of each RNA sample was confirmed by calculating the ratio of optical density at 260∶280 nm. Reverse transcription was performed with random primers on 1.25 μg of total RNA in a final reaction volume of 50 microliters using the High-Capacity cDNA Archive Kit (Applied Biosystems, Foster City, CA, USA). Quantitative real-time PCR was carried out on the BioRad iCycler iQ system using iQ^TM^ SybrGreen Supermix (BioRad, Hercules, CA, USA) as specified by the manufacturers' instructions, and the fluorescence threshold values were calculated using iCycle iQ system software. Human Bax, BcL-2, cytochrome c, caspase-9, and GAPDH primer sequences are available in the public RTPrimerDB database (http://medgen.UGent.be/rtprimerdb/). Melting curves were also acquired and analyzed to ensure specificity of the reaction. Each sample was run in duplicate and normalized to the GAPDH transcript content. The fold change in mRNA levels was calculated, with corrections for the housekeeping gene GAPDH. Separate control experiments using serial dilutions of cDNA demonstrated that the efficiencies of each target gene and GAPDH amplification were similar, hence validating the use of comparative Ct method to determine relative expression of genes of interest (data not shown).

#### Western blot analysis

After treatment, lysates of ARPE-19 cells were obtained, and total protein was extracted in protein lysis buffer M-PER (Pierce, Rockford, IL, USA) and quantified by a Detergent Compatible protein assay (Bio-Rad, Hercules, CA, USA). Protein extracts (20 to 40 μg) were denatured in Laemmli's sample buffer, followed by boiling for 5 minutes, and then resolved on a 4 to 20% Tris-glycine gel. After electrophoresis (120 V for 2 hours), proteins were transferred in 1X transfer buffer [25 mmol/L Tris, 192 mmol/L glycine, 0.1% SDS, and 20% methanol (pH 8.4)] to a Hybond-ECL nitrocellulose membrane (Amersham Biosciences, Piscataway, NJ, USA) using constant current (350 mA for 45 minutes). Membranes were blocked in 5% nonfat dry milk – TBS solution for 1 hour at room temperature. Blots were incubated overnight at 4°C with primary antibodies against Bax, cytochrome c, caspase-9, Bcl-2, and GAPDH. Membranes were washed three times with TBS-Tween 20, then incubated with horseradish peroxidase – linked donkey anti-rabbit anti-mouse antibody for 2 hours at room temperature, and finally washed three times in TBS-Tween 20. Immunoreactive bands were determined by exposing the nitrocellulose blots to a chemiluminescent solution and exposing to X-Omat AR film (Eastman Kodak Co., Rochester, NY, USA). Three independent experiments were performed in triplicate.

### Cadaveric human eye staining affinity assay

In the present paper, the safety profile of both dyes: AcV and MetB were evaluated; as the use of such dyes in chromovitrectomy was not reported before (*source: Medline, December 2012*), a staining affinity experiment in cadaveric eyes was performed in order to evaluate the capacity of ILM staining after dye exposure. Fresh cadaveric eyes (within 24 hours after death) were obtained from the Sorocaba Eye Bank after previous IRB approval and permission from the Transplant State Center of São Paulo as well as informed consent of the family members of the donor that stated they were informed about the purposes of the study. The cornea was removed at the eye bank for transplant purposes and the remaining structures of the globe were used for the study. An open sky vitrectomy was performed with Constellation Vision System (Alcon, Fort Worth, TX, USA). After removal of the vitreous, 2 ml of 0.05 mg/ml AcV or MetB were applied over the macular region for 3 minutes and then washed away with balanced salt solution. Photographs of the posterior pole were than obtained for documentation purposes; BriB and ICG were not evaluated in this part of the study since it is well known that they have a good affinity to the ILM and because they are in current use in vitreoretinal surgeries.

### Statistical analyses

All experiments were performed three times, showing reproducible results. Statistical analyses were performed using GraphPad Prism 5 software (GraphPad, La Jolla, CA, USA). Data are expressed as the mean ± SEM of percentage of cell viability/toxicity with respect to control. Statistical comparisons were performed using one-way analysis of variance followed by Tukey's post hoc test for multiple comparisons. Values of P<0.05 were considered statistically significant.

## Results

### Effect of BriB, MetB, AcV and ICG dyes on cell viability

The toxicity range of the dyes was assessed by treating the cells with the four different dyes at different concentrations (1, 0.5, 0.25, 0.05, and 0.005 mg/ml) for 3 minutes or 30 minutes. Cell viability was determined by the MTS reduction assay, cell counts, and LDH release.

**Figure 1:**
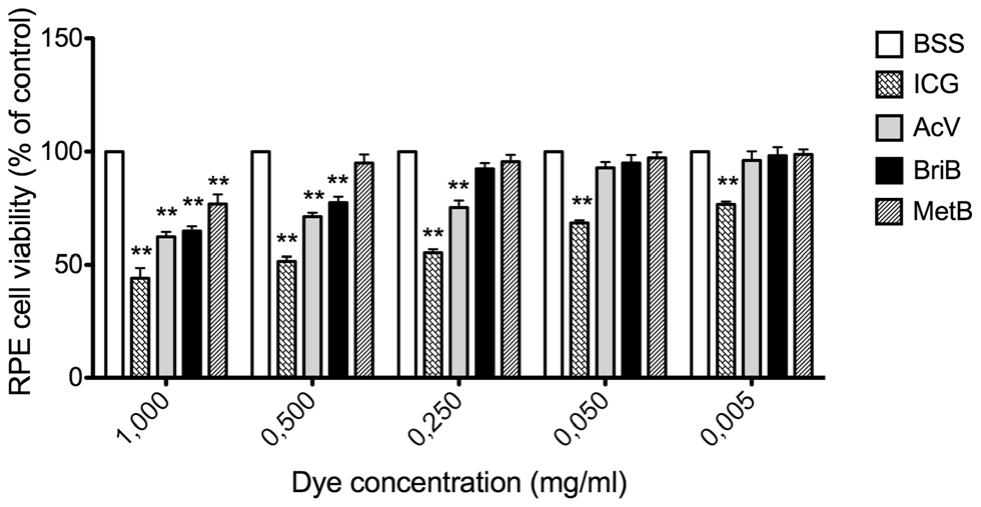
Effect of different doses of indocyanine green (ICG), acid violet (AcV), brilliant blue (BriB) and methyl blue (MetB) on ARPE-19 cells viability assessed by MTS cell assay. Cells were exposed to dyes for 3 minutes. Bars correspond to means of three independent experiments. Data are expressed as percentage of control, and the results shown are mean results ± SEM of three independent experiments run in triplicate on cultured cells. Statistical significance is indicated by **P<0.01 and for comparison with the control (BSS solution).

**Figure 2:**
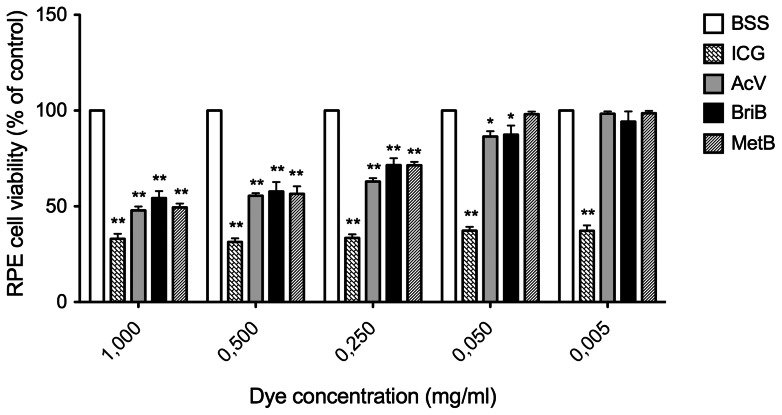
Effect of different doses of indocyanine green (ICG), acid violet (AcV), brilliant blue (BriB) and methyl blue (MetB) on ARPE-19 cells viability determined by MTS cell assay after 30 minutes of exposure to dyes. Bars correspond to means of three independent experiments. Data are expressed as percentage of control, and the results shown are mean results ± SEM of three independent experiments run in triplicate on cultured cells. Statistical significance is indicated by *P<0.05, **P<0.01 for comparison with the control (BSS solution).

As shown in Figure 1, all concentrations tested for ICG during 3 minutes of exposure significantly decreased cell viability when compared to control (BSS). This effect was dose-dependent (Table 1). Moreover, treatment with this dye for 30 minutes dramatically reduced the viability when compared to control (Figure 2 Table 1). ICG (1 mg/ml) for 3 minutes decreased cell viability by approximately 57% (43.1±3.3%; P<0.01) whereas 0.5 mg/ml and 0.25 mg/ml ICG decreased cell viability by approximately 49% (51.6%±3.5%; P<0.01, 55.2%±2.7%; P<0.01) and concentrations of 0.050 mg/ml and 0.005 mg/ml by approximately 38% (61.9%±4.2%; P<0.01, 66.3%±3.9%; P<0.01) (Figure 1). However, after 30 minutes of treatment the decrease in cell viability was ∼70% for all the assayed doses (Figure 2 and Table 1).

**Table 1:** Effect of vital dyes on retinal pigment epithelial cell viability, at different dye concentrations and exposure times.

Time exposure	Dyes	Concentrations (mg/ml)				
		1	0.5	0.25	0.05	0.005
		(Mean ± SD)	(Mean ± SD)	(Mean ± SD)	(Mean ± SD)	(Mean ± SD)
**3 minutes**	**BSS**	100±0	100±0	100±0	100±0	100±0
	**ICG**	43.1±3.3 **	51.6±3.5 **	55.2±2.7 **	61.9±4.2 **	66.3±3.9 **
	**AcV**	64.1±1.5 **	74.5±1.1 **	76.4±5.8 **	95.5±7	97.6±4.4
	**BriB**	64.8±3.6 **	77.4±4.5 **	94.4±4.9	94.9±6.2	98.2±6.5
	**MetB**	76.1±4.3 **	94.9±6.5	95.6±5.2	97.3±4.3	98.8±3.9
**30 minutes**	**BSS**	100±0	100±0	100±0	100±0	100±0
	**ICG**	33.0±4.6 **	31.4±3.3 **	33.5±3.3 **	37.2±3.6 **	37.2±5 **
	**AcV**	56.5±2.3 **	63.2±3.1 **	69.4±2.3 **	87.3±2.9 *	96.8±4.8
	**BriB**	62.3±3.1 **	72.8±5.1 **	81.6±6.1 **	87.4±8.3 *	94.2±9.3
	**MetB**	49.4±3.5 **	49.9±2.8 **	71.4±3 **	98.2±2.3	98.6±2.1

Analysis of variance (ANOVA) –Tukey test Observations:

I – Lower the values higher the dye toxicity.

II – Values are means of at least 3 independent experiments.

III – In regard to the P values presented in this table, there was a significant difference between the dyes with all concentrations and exposure times (* P<0.05, ** P<0.01) when compared with non-treated control cells.

Exposure to 1 and 0.5 mg/ml BriB for 3 minutes showed a statistically significant reduction in cell viability of ∼35% and 23% respectively (64.8%±3.6%; P<0.01, 77.4%±4.5%; P<0.01). However, doses of 0.25, 0.05 and 0.005 mg/ml, did not modify cell viability (Figure 1, Table 1). When cells were exposed to this dye for 30 minutes, cell viability decreased by ∼37% and 27% at concentrations of 1 to 0.05 mg/ml respectively (62.3%±3.1%; P<0.01, 72.8%±5.1%; P<0.01) and by ∼18% and 13% (81.6%±6.1%; P<0.01, 87.4%±8.3%; P<0.05) at concentrations of 0.25 and 0.05 respectively (Figure 2, Table 1). BriB (0.005 mg/ml) did not modify cell viability.

Our data also show that AcV for 3 minutes induced a decrease in cell viability at doses higher than 0.05 mg/ml (64.1%±1.5%; P<0.01, 74.5%±4.5%; P<0.01, 76.4%±5.8%; P<0.01) whereas it had no effect at concentrations of 0.05 mg/ml and 0.005 mg/ml (Figure 1, Table 1). However, exposure to AcV for 30 minutes decreased cell viability at doses over 0.005 mg/ml (Figure 2, Table 1).

We found that MetB, decreased cell viability by ∼24% only at 1 mg/ml after 3 minutes of exposure (76.1.3%±4.3%; P<0.01). Therefore, MetB for 3 minutes had the least toxic profile of all dyes. In contrast, MetB at concentrations higher than 0.05 mg/ml for 30 minutes significantly decreased cell viability (Figures 1 and 2, Table 1).

**Figure 3:**
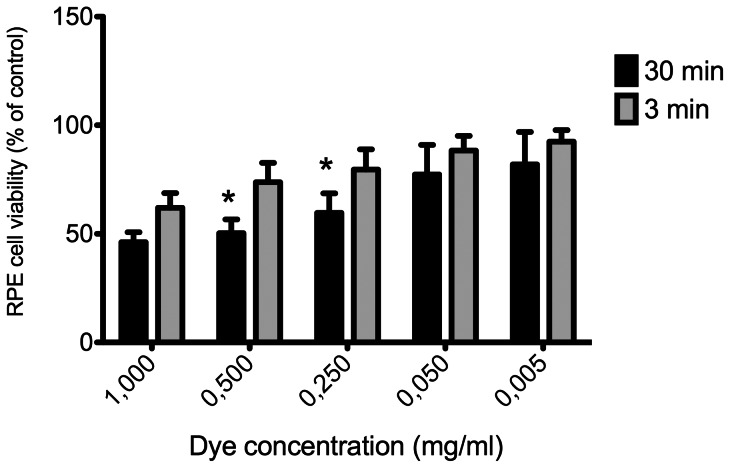
Combined results of effect of vital dyes on ARPE-19 cells. The effects of dyes on cell viability were combined for each concentration and compared between 3 and 30 minutes of exposure. Bars correspond to means of three independent experiments. Data are expressed as percentage of control, and the results shown are mean results ± SEM of three independent experiments run in triplicate on cultured cells. Statistical significance is indicated by *P<0.01 for comparison with the control.

When exposure time was evaluated as an isolated factor, it was shown that for the highest (1 mg/ml) or lowest (0.005 mg/ml) dye concentrations, RPE cell viability did not differ significantly with 3 versus 30 minutes of exposure. However, at intermediate concentrations (0.5 or 0.25 mg/ml), RPE cell viability was significantly reduced with 30 minutes of exposure (Figure 3).

**Figure 4:**
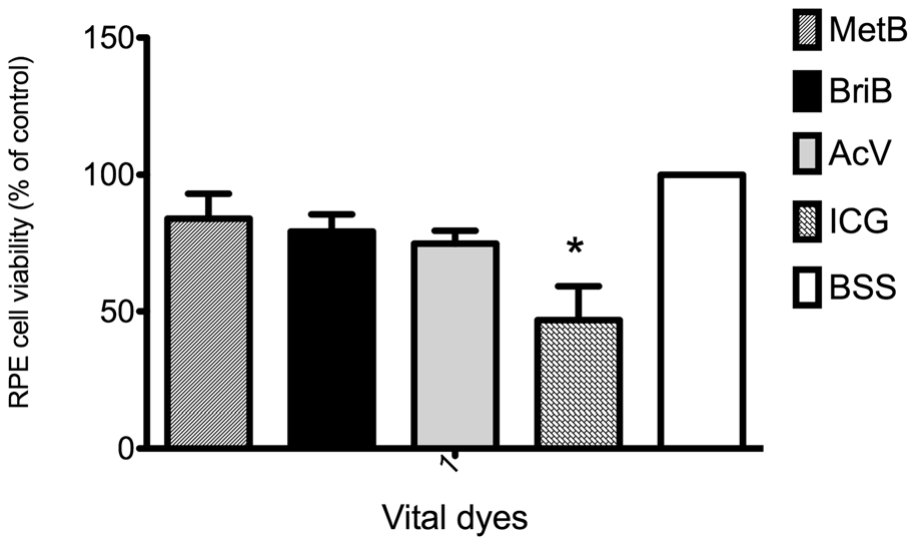
Combined results of effect of vital dyes on ARPE-19 cells viability. The effects of dyes on cell viability were combined for all concentrations and both exposure times and the dyes compared. Bars correspond to means of three independent experiments. Data are expressed as percentage of control, and the results shown are mean results ± SEM of three independent experiments run in triplicate on cultured cells. Statistical significance is indicated by *P<0.01 for comparison with the control (BSS solution).

In regard to the dye itself, when all values were analyzed independently of concentration or exposure time, the blue dyes (BriB, MetB and AcV) did not differ from the control (BSS). On the other hand, ICG caused a significant decrease in RPE cell viability compared to the control or other dyes (Figure 4). Comparable results were observed for cell viability measured by cell counts (data not shown).

**Figure 5:**
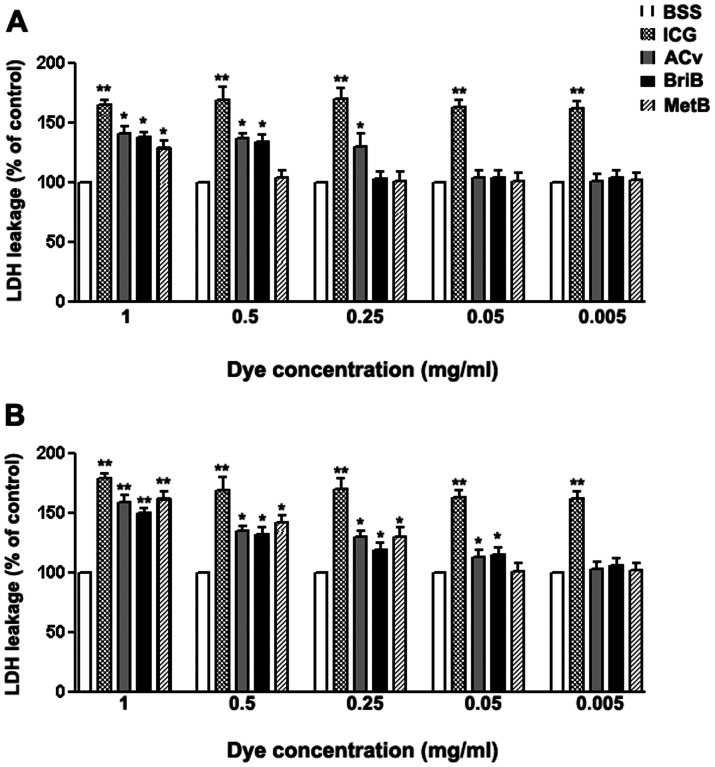
ARPE-19 cell viability assayed by measuring LDH release following dyes treatment. The cells were treated with 0.05 mg/ml indocyanine green (ICG), acid violet (AcV), brilliant blue (BriB) and methyl blue (MetB) for 3 minutes (**A**) or 30 minutes (**B**) in BSS. Data are expressed as percentage of control, and shown are mean results ± SEM of three independent experiments run in triplicate on cultured cells. Statistical significance is indicated by *p<0.05 and **p<0.01 versus control.

The permeability and integrity of cell membranes were determined using the LDH-release kit. As shown in Figure 5A and B, ARPE-19 cells exposed to ICG exhibited a significant increase in LDH release when compared to the control (p<0.01). This increase was independent of dose and exposure time. Our data also showed a significant increase in LDH release when cells were treated for 3 minutes with AcV (1, 0.5 and 0.25 mg/ml), BriB (1, and 0.5 mg/ml), and MetB (0.5 mg/ml) (p<0.05) (Figure 5A, Table 2). Exposure to AcV and BriB for 30 minutes increased LDH release at doses over 0.05 mg/ml, whereas for MetB, the observed LDH release was at doses over 0.005 mg/ml (p<0.01 and p<0.05 respectively). Overall the cytotoxic activity of these three dyes was substantially more potent at 1 mg/ml (Figure 5B, Table 2) and ICG showed the most potent and rapid cytotoxic effect of all dyes studied.

**Table 2:** Cytotoxic effect of vital dyes on retinal pigment epithelial cells determined by LDH assay.

Time exposure	Dyes	Concentrations (mg/ml)				
		1	0.5	0.25	0.05	0.005
		(Mean ± SD)	(Mean ± SD)	(Mean ± SD)	(Mean ± SD)	(Mean ± SD)
**3 minutes**	**BSS**	100±0	100±0	100±0	100±0	100±0
	**ICG**	162.50±3.12 **	168.75±12.51 **	167.23±6.25 **	163.12±5.62 **	181.71±5.83 **
	**AcV**	139.72±6.19 *	138.50±3.13 *	136.61±11.8 *	112.40±3.17	101.87±5.12
	**BriB**	136.21±2.91 *	135.17±4.71 *	103.58±6.15	110.31±6.30	113.02±7.01
	**MetB**	130.00±7.31 *	102.43±7.10	101.10±9.31	106.20±9.01	105.60±6.01
**30 minutes**	**BSS**	100±0	100±0	100±0	100±0	100±0
	**ICG**	178.01±3.12 **	168.61±5.97 **	167.82±3.51 **	162.21±6.03 **	161.53±2.71 **
	**AcV**	162.00±5.87 **	134.52±2.87 *	130.17±3.43 *	112.61±6.02	99.37±7.15
	**BriB**	150.41±5.78 **	125.00±6.23 *	125.50±6.23 *	112.00±6.1	100.31±6.11
	**MetB**	162.42±6.13 **	143.15±4.61 *	127.70±12.3 *	99.81±8.12	101.23±7.03

Analysis of variance (ANOVA) –Tukey test Observations:

I – Higher the values higher the dye toxicity.

II – Values are means of at least 3 independent experiments.

III – In regard to the P values presented in this table, there was a significant difference between the dyes with all concentrations and exposure times (* P<0.05, ** P<0.01) when compared with non-treated control cells.

### Regulation of Bax and Bcl-2 expression by vital dyes in ARPE-19 cells

**Figure 6:**
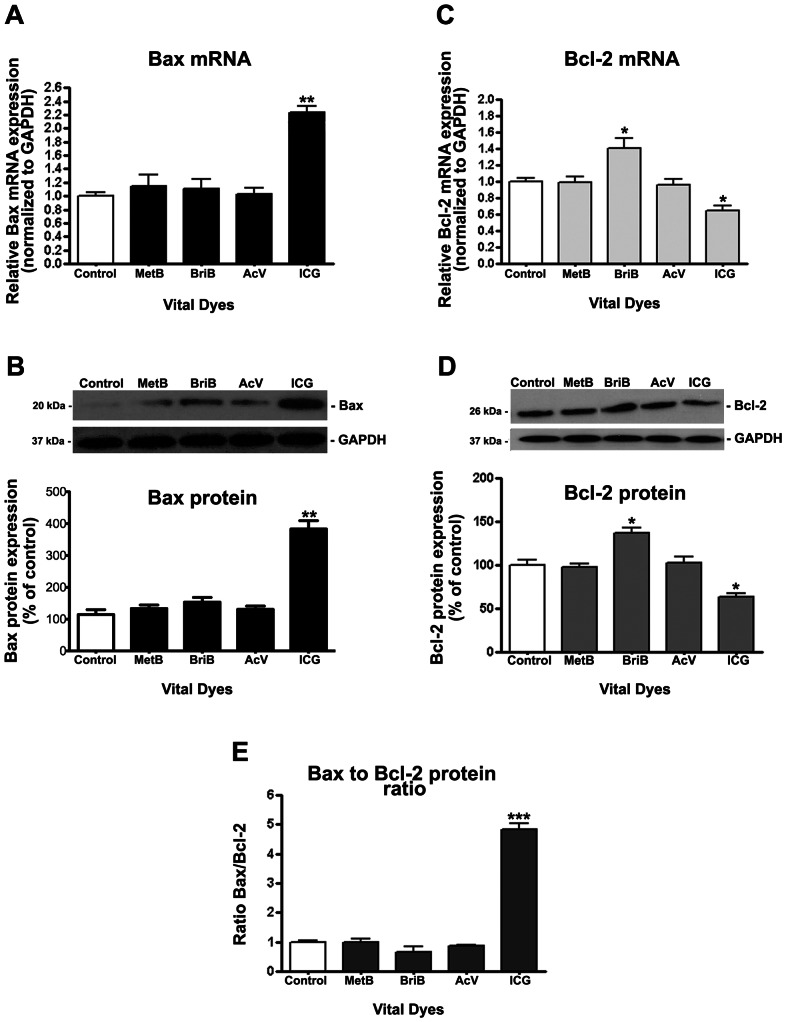
Bax/Bcl-2 ratio in ARPE-19 cells following treatment with vital dyes. Confluent ARPE-19 cells were treated with 0.05 mg/ml indocyanine green (ICG), acid violet (AcV), brilliant blue (BriB) and methyl blue (MetB) for 3 minutes in BSS. Total RNA and proteins were extracted to assess Bax (**A**) and Bcl-2 (**C**) mRNA expression and Bax (**B**) and Bcl-2 (**D**) protein expression by real-time PCR and Western blot respectively. GAPDH was used as the internal control. Representative Western blot gel (*Top*) of proapoptotic protein Bax (**B**) and antiapoptotic protein Bcl-2 (**D**). The numbers to the left are molecular weights in kilodaltons (kDa). *Bottom*: average densitometry results from three independent experiments. (**E**) Bax/Bcl-2 protein ratio. Data are mean ± SE and represent the average results of 3 independent experiments run in duplicate. * is p<0.05, ** is p<0.01 and *** is p<0.001 versus control.

In many systems, members of the Bcl-2 family modulate apoptosis, with the Bax/Bcl-2 ratio serving to as a rheostat to determine cell susceptibility to apoptosis [32]. Dysregulated expression of Bax and Bcl-2 by RPE may be involved in the cell susceptibility to apoptosis. Therefore, based on the observations obtained for cell viability, it was explored whether ICG induces apoptosis through Bax overexpression in ARPE-19 cells. With the use of real-time PCR and Western blot analysis, it was investigated the expression of Bax and Bcl-2 in ARPE-19 cells treated with or without 0.05 mg/ml BriB, MetB, AcV, and ICG for 3 minutes. Our results revealed that ICG strongly increases Bax expression at the mRNA and protein levels (Figure 6A, B). Bax transcription increased by ∼2.2-fold compared with untreated cells (2.23±0.08 versus 1.0±0.03, p<0.01) (Figure 6A) whereas Bax protein increased by ∼3.4-fold (383.3%±26.03 versus 114.3±15.35%, p<0.01) (Figure 6B). BriB, MetB and AcV revealed a slightly increase in Bax expression, but it was not significantly when compared to untreated cells (Figure 6A, B).

The results also revealed a significant decrease in the expression of Bcl-2 at the mRNA and protein levels when cells were treated with ICG. Bcl-2 mRNA and protein expression was decreased by ∼37% (0.63±0.13 versus 1.0±0.12, p<0.05 and 100.0±4.3% versus 163.2±2.80%, p<0.05 respectively) (Figure 6C, D). These results indicate a robust ∼ 4.5-fold increase in Bax/Bcl-2 protein ratio (4.75±12.3 versus 1.0±0.12, p<0.001) which may switch the balance in favor of apoptosis stimulation over inhibition (Figure 6E). MetB and AcV failed to modify Bcl-2 expression. However, BriB revealed a discrete but significant increase in Bcl-2 expression at the transcription (1.38±0.18 versus 1.0±0.09, p<0.05) and transduction (143.7±0.12% versus 100.0±0.10%, p<0.05) levels (Figure 6C, D). Overall, the Bax-to Bcl-2 protein ratio was slightly decreased but not significantly when compared to untreated cells (Figure 6E).

### ICG induces apoptosis via release of cytochrome c and activation of caspase-9

**Figure 7:**
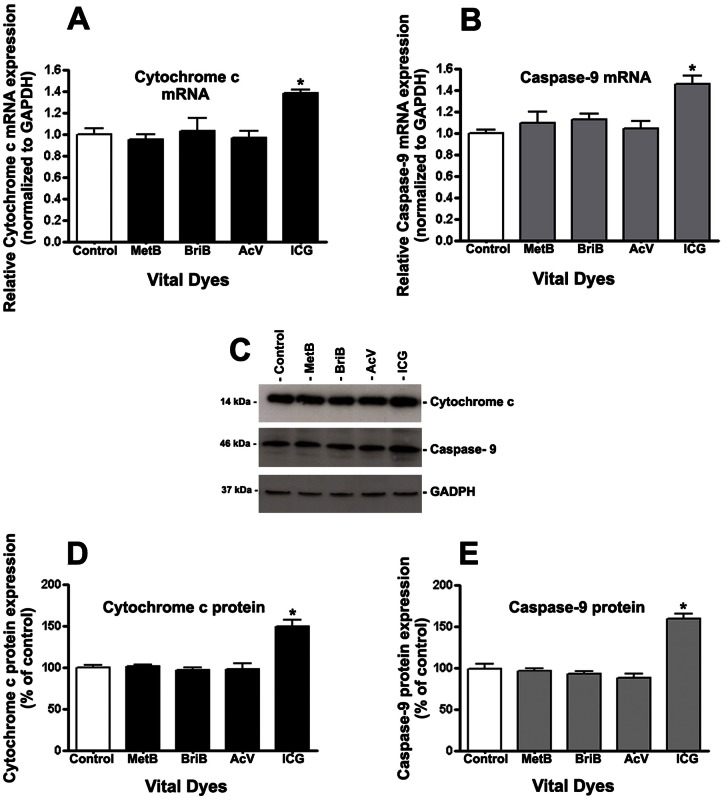
Cytochrome c and Caspase-9 were upregulated by ICG in ARPE-19 cells. Cytochrome c (**A and D**) and caspase-9 (**B and C**) mRNA and protein expression in response to 0.05 mg/ml BriB, MetB, AcV, and ICG treatment for 3 minutes. Total RNA and proteins were extracted to assess cytochrome c (**A**) and caspase-9 (**C**) mRNA expression and Cytochrome c (**B**) and Caspase-9 (**D**) protein expression by real-time PCR and Western blot respectively. GAPDH was used as the internal control. Representative Western blot gels (**C**). The numbers to the left are molecular weights in kilodaltons (kDa). Average densitometry results of three independent experiments (**D, E**). Data are mean ± SE and represent the average results of 3 independent experiments run in duplicate. * is p<0.05 versus control.

It has been shown that Bax high levels induces apoptosis by increasing the release of cytochrome c and caspase activation *in vivo* and *in vitro* [33]. Therefore, we studied whether cytochrome c and caspase-9 are involved in IGC-induced apoptosis by examining the protein expression of cytochrome c and caspase-9 by real-time PCR and Western blots. The results revealed a significant increase in the expression of cytochrome c and caspase-9 at the mRNA and protein levels when cells were treated with ICG. The cytochrome c mRNA and protein expression were increased by ∼40% (1.0±0.06 versus 1.4±0.02; P<0.05) and ∼50% (100.01±0.86% vs. 152.17±8.67%; P<0.05) respectively (Figure 7A, D). The results also revealed that ICG increases caspase-9 expression at the mRNA and protein levels (Figure 7B, E). Caspase-9 transcription increased by ∼1.6-fold compared with untreated cells (1.0±0.06 versus 1.57±0.12; p<0.05) (Figure 7B) whereas the protein increased by ∼75% (100.05±2.34% 176.82±7.61%; p<0.05) (Figure 7E). As expected, MetB, BriB, and AcV failed to modify cytochrome c and caspase-9.

### Cadaveric human eye staining affinity assay

**Figure 8:**
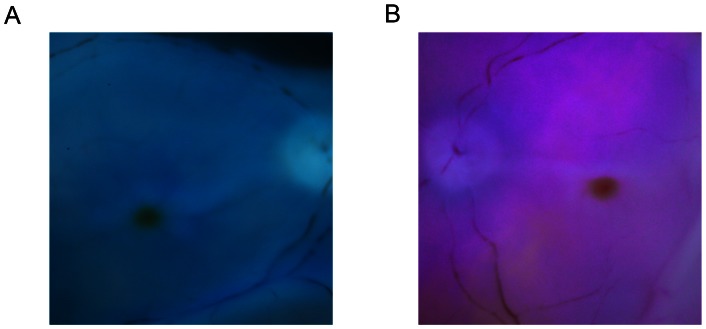
Internal limiting membrane (ILM) staining affinity for methyl blue (MetB) and acid violet (AcV) in human eyes. Cadaveric human eyes were vitrectomized and exposed to 0.05 mg/ml MetB or AcV for 3 minutes. MetB provided a very good bluish staining of the ILM (**A**). AcV, on the other hand, provided a violet/pink staining, with a good visualization of the ILM (**B**).

After 3 min of dye exposure in vitrectomized cadaveric eyes, 0.05 mg/ml of MetB and AcV had a good ILM staining capacity due to a good contrast with the red unstained retina subjacent to the ILM, which facilitated the surgical peeling of the ILM (Figure 8).

## Discussion

Since the introduction of membrane staining, an ongoing debate about the safety of vital dye has emerged. The use of vital dyes in vitrectomy facilitates intraoperative surgical procedures such as ILM or epiretinal membrane (ERM) peeling. The two dyes available for chromovitrectomy, ICG and TB, may lead to complications in macular surgery, and they may migrate to the subretinal space and produce alterations in the RPE and campimetric and papillary defects. In particular ICG is supposed to have a dose- and illumination-dependent toxicity in photoreceptors, retinal ganglion cells and the RPE, even at clinically used concentrations [34], [35]. Moreover, controlled clinical trials evaluating ICG-assisted ILM-peeling have raised concerns about its use [36]–[38]. Therefore, in this study, a detailed *in vitro* toxicity investigation of the dyes MetB, BriB, AcV and ICG was done.

Five different dye concentrations (1, 0.5, 0.25, 0.05 and 0.005 mg/ml) and two exposure times (3 and 30 min) were selected to represent the whole gamut of possible dye dilutions and injection techniques that can be performed by surgeons during chromovitrectomy. For example, by one hand, if ICG is prepared at a concentration of 0.25 mg/ml and injected in an air-filled vitreous cavity, 0.25 mg/ml is the final concentration in contact with the retina. One the other hand, if the dye is injected in a BSS-filled vitreous cavity, the final concentration will be divided by 4 due to the 4-ml volume of the vitreous cavity. This subject was previously discussed in a review paper where some recommendations were made to avoid retinal toxicity when ICG is used: use concentrations not higher than 0.05 mg/ml, in fluid-filled eyes, with short exposure times, using iso-osmolar solutions, and avoid proximal or prolonged endoillumination of stained tissue or even to avoid the ICG use in macular hole surgery due to its toxicity profile [34].

AcV belongs to the cyanine dyes, characterized by the presence of a 5-methine bridge linking two nitrogen-containing heterocyclic rings. Cyanine dyes are part of a larger group called polymethine dyes, which have one or more -CH =  (methine) groups. Cyanine dyes are highly colored, organic compounds, which were first synthesized over a century ago. They have been mostly used as sensitizers in photography or textile dyeing. In the present study, AcV showed a safe profile up to 0.5 mg/ml after 3 minutes of exposure. After 30 minutes, BriB and AcV did not induce cell damage up to 0.005 mg/ml. Based on these results, a short incubation time and low concentration should be chosen when using the cyanine blue vital dyes.

MetB is a family of organic compounds that are mainly used as dyes. Depending on the number of attached methyl groups, the color of the dye can be altered. The term methyl blue encompasses three compounds that differ in the number of methyl groups attached to the amine functional group. Haritoglou et al. [39] and Thaler et al. [40] demonstrated the staining ability and in vivo safety of MetB in human donor and rat's eyes. Herein, we observed MetB to be nontoxic at concentrations up to 0.25 mg/ml. Moreover, in contrast to all other dyes, which showed remarkable toxic effects, MetB was safe up to 0.05 mg/ml. These findings suggest that MetB may be a quite safe stain for surgical use in chromovitrectomy.

Brilliant Blue G (BBG), also known as Coomassie or Acid Blue, is a blue biostain certified as a safe food additive in Europe and may be used as a marker for cardiovascular and neurologic disease proteins. The safety profile of BBG was investigated by Enaida et al., and later evaluated by other groups in pre-clinical and clinical experiments [41]. BBG emerged as the first truly safe alternative for ICG and infracyanine green (IfCG) in chromovitrectomy due to its remarkable affinity to the ILM, but limited toxicity data on BBG application during chromovitrectomy warrants further investigations.

In the present study, the safety profiles of ICG, BriB, MetB and AcV were compared. The safest dye with regard to RPE cells was MetB followed by BriB and AcV. ICG significantly reduced cell viability after 3 minutes of exposure at all concentrations. BriB and MetB were safe at concentrations up to 0.25 mg/ml and 0.5 mg/ml respectively, while AcV was not toxic up to 0.5 mg/ml after 3 minutes of exposure. However, when the cells were exposed to dyes for 30 minutes, all dyes showed remarkable toxic effects, except for MetB at the lower concentrations (0.05 and 0.005 mg/ml) and BriB or AcV (0.005 mg/ml).

On the basis of our viability data and since it was shown that residual ICG can be found months after surgery [42], the evaluation of apoptosis in retinal toxicity studies of this dye has become an important issue. Therefore, the present study also evaluated the link between ICG exposure and apoptosis in RPE cells by studying Bax and Bcl-2, two members of the Bcl-2 protein family, which are involved in controlling apoptosis events. A high level of the anti-apoptotic protein Bcl-2, inhibits apoptosis by preventing cytochrome c release while a high level of the pro-apoptotic protein Bax, induces apoptosis by binding to the mitochondrial membrane allowing release of cytochrome c [33]. This paper shows that ICG remarkably induced Bax expression and downregulated Bcl-2 expression, which in turn increased the Bax/Bcl-2 ratio after 3 minutes of exposure at 0.05 mg/ml, a common concentration still in use by some surgeons. In contrast, BriB, MetB and AcV did not modify the Bax/Bcl-2 ratio. Interestingly, BriB upregulated the anti-apoptotic protein Bcl-2, pointing out a possible protective role of this dye. The protective potential effect of BriB was first demonstrated by Kimbler et al. [43]. The authors showed that BBG could inhibit the P2X7 and therefore reduce the brain damage after traumatic neurological injury. After that some ophthalmologists were claiming that BriB could also be a protective agent after intravitreal use in vitreoretinal surgeries. However, further studies should be done to prove this hypothesis.

In agreement with our data, Balaiya et al. also showed that ICG but not BriB induces apoptosis in retinal cells [44]. However to the best of our knowledge, the apoptosis signaling pathway induced by ICG has not yet been previously reported (source: Medline, March 2013). There are two major apoptosis signaling pathways; the death receptor (extrinsic) pathway and the mitochondria-mediated (intrinsic) pathway; the intrinsic pathway is initiated by several stimuli, which induce permeabilization of the outer mitochondrial membrane, activating the mitochondrial pathway; the mitochondrial pathway is engaged by the release of apoptogenic factors such as cytochrome c from the mitochondrial intermembrane space into the cytosol. This release of cytochrome c into the cytosol triggers caspase-9 activation [45]. The present study revealed that ICG treatment resulted in an upregulation of cytochrome c and caspase-9 expression, which may culminate in caspase-dependent cell death. Whether and how ICG disrupts mitochondrial integrity by targeting the Bcl-2 family proteins is not yet characterized. Therefore, studies targeting the Bcl-2 family remain an interesting possibility to be investigated.

The present paper does not address the important issue about the toxicity of dye-light interaction. This was part of a separate investigation where our group evaluated six dyes (trypan blue, brilliant blue, bromophenol blue, indigo carmine, light green and fast green) [46]. After dye exposure the cells where illuminated with two vitrectomy light sources, high-brightness xenon and mercury vapor for 10 min. No decreased on cell viability was observed when compared to the control BSS. The lack of toxicity could be explained by the relatively short time exposure, but the potential toxicity of dye-light interaction is yet to be determined.

It is important to state that the results of this paper should not be extrapolated to entire retinal toxicity induced by the dyes studied. The current study evaluated dye toxicity and induction of apoptosis only in a RPE human cell line (ARPE-19). Besides this drawback, this design of study is necessary since direct dye-RPE contact may occur during macular hole surgery and it may induce RPE defects and vision loss. Experiments using other cell lines, such as R28 and ganglion cells are currently under investigation and should be important to address the dye toxicity profile in retinal inner layers.

In summary, ICG was shown to be toxic at all concentrations tested with two exposure times, and it was the only dye that substantially increased the Bax/Bcl-2 protein ratio, shedding light on the molecular mechanisms of ICG-induced apoptosis. The safest dye was MetB, followed by BriB and AcV. Higher concentration and longer exposure time increased toxicity profile with all tested dyes. BriB was not toxic at concentrations lower than 0.25 mg/ml and may have a protective effect over RPE cells since it increased the expression of Bcl-2. However this *in* vitro observation deserves further investigation to support this possible benefit for use during chromovitrectomy in humans.
